# Beta‐blockers withdrawal in patients with heart failure with preserved ejection fraction and chronotropic incompetence: Effect on functional capacity rationale and study design of a prospective, randomized, controlled trial (The Preserve‐HR trial)

**DOI:** 10.1002/clc.23345

**Published:** 2020-02-19

**Authors:** Patricia Palau, Julia Seller, Eloy Domínguez, Inés Gómez, José María Ramón, Clara Sastre, Rafael de la Espriella, Enrique Santas, Gema Miñana, Francisco J. Chorro, José Ramón González‐Juanatey, Julio Núñez

**Affiliations:** ^1^ FISABIO. Universitat Jaume I Castellón Spain; ^2^ Cardiology Department Hospital de Denia Alicante Spain; ^3^ Cardiology Department Hospital Clínico Universitario de Santiago de Compostela, Santiago de Compostela A Coruña Spain; ^4^ CIBERCV Madrid Spain; ^5^ Cardiology Department Hospital Clínico Universitario, INCLIVA. Universitat de València Valencia Spain

**Keywords:** chronotropic incompetence, exercise capacity, heart failure with preserved ejection fraction, quality of life

## Abstract

**Background:**

The pathophysiology of heart failure with preserved ejection fraction (HFpEF) is complex and multifactorial. Chronotropic incompetence (ChI) has emerged as a crucial pathophysiological mechanism. Beta‐blockers, drugs with negative chronotropic effects, are commonly used in HFpEF, although current evidence does not support its routine use in these patients.

**Hypothesis:**

We postulate beta‐blockers may have deleterious effects in HFpEF and ChI. This work aims to evaluate the short‐term effect of beta‐blockers withdrawal on functional capacity assessed by the maximal oxygen uptake (peakVO2) in patients with HFpEF and ChI.

**Methods:**

This is a prospective, crossover, randomized (1:1) and multicenter study. After randomization, the clinical and cardiac rhythm will be continuously registered for 30 days. PeakVO2 is assessed by cardiopulmonary exercise testing (CPET) at 15 and 30 days in both groups. Secondary endpoints include quality of life, cognitive, and safety assessment. Patients with stable HFpEF, functional class New York Heart Association (NYHA) II‐III, chronic treatment with beta‐blockers, and ChI will be enrolled. A sample size estimation [alfa: 0.05, power: 90%, a 20% loss rate, and delta change of mean peakVO2: +1.2 mL/kg/min (SD ± 2.0)] of 52 patients is necessary to test our hypothesis.

**Results:**

Patients started enrolling in October 2018. As January 14th, 2020, 28 patients have been enrolled. It is projected to enroll the last patient at the end of July 2020.

**Conclusions:**

Optimizing therapy that improves functional capacity remains an unmeet priority in HFpEF. Deprescribing beta‐blockers in patients with HFpEF and ChI seems a plausible intervention to improve functional capacity. This trial is an attempt towards precision medicine in this complex syndrome.

**Trial registration:**

http://clinicaltrials.gov: NCT03871803.

**Abbreviations**
ChI: chronotropic incompetence
CPET: cardiopulmonary exercise testing
HF: heart failure
HFpEF: heart failure with preserved ejection fraction
HFrEF: heart failure with reduced ejection fraction
NYHA: New York Heart Association
PeakVO2: peak exercise oxygen uptake
QoL: quality of life

## 1 INTRODUCTION

Heart failure (HF) with preserved ejection fraction (HFpEF) is a heterogeneous syndrome that is the predominant form of HF in western countries.^1^, ^2^, ^3^, ^4^ Chronotropic incompetence (ChI), defined as the inability to increase heart rate during exercise adequately, is commonly present (ranging from 20 to 75%) in HFpEF patients.^5^, ^6^, ^7^, ^8^, ^9^, ^10^, ^11^ Furthermore, ChI has been proposed as a pathophysiologic mechanism associated with poorer outcomes and decreased functional capacity in a subgroup of patients with HFpEF.^8^, ^12^, ^13^

Data from current registries show a high proportion (ranging from 50% to 80%) of beta‐blockers prescription in HFpEF patients regardless of the heart rhythm.^3^, ^14^ Nevertheless, there is no well‐established evidence endorsing the effect of beta‐blockers. For instance, recent studies suggested that patients with an ejection fraction of 50% or greater did not see any benefits from receiving beta‐blockers.^15^, ^16^, ^17^ Even more, the evidence is missing stratifying patients with HFpEF across ChI status.

When ChI is present, beta‐blockers may have negative effects on functional capacity and other surrogates of the disease severity by exacerbating the ChI. Thus, we hypothesize that deprescribing beta‐blockers in this particular scenario will translate into an improvement in short‐term maximal functional capacity. The purpose of this randomized controlled study is to evaluate the short‐term effects of beta‐blockers withdrawal on the functional capacity, cognitive function, and quality of life (QoL) in patients with HFpEF and documented ChI.

The primary endpoint of the study is absolute and relative changes in peak oxygen uptake (peakVO2) at 15‐day after the intervention. The secondary endpoints are: (a) 15‐day absolute changes in cognitive function assessed by the Mini‐Mental State Examination (MMSE) and Montreal Cognitive Assessment (MoCa); (b) 15‐day absolute changes in echocardiogram parameters (E/E' ratio and left atrial volume index); (c) 15 days absolute changes in QoL assessed by Minnesota Living With Heart Failure Questionnaire (MLHF); and (d) 15 days absolute and relative changes in prognostic biomarkers (N‐terminal pro‐B‐type natriuretic peptide ‐NT‐proBNP‐ and serum carbohydrate antigen 125‐CA125‐).

Safety endpoints include the composite event of the total number of episodes of acute HF hospitalizations, total episodes of worsening HF not requiring hospitalization or mortality at 6 months.

## 2 METHODS

### 2.1 Study design

**Figure 1:**
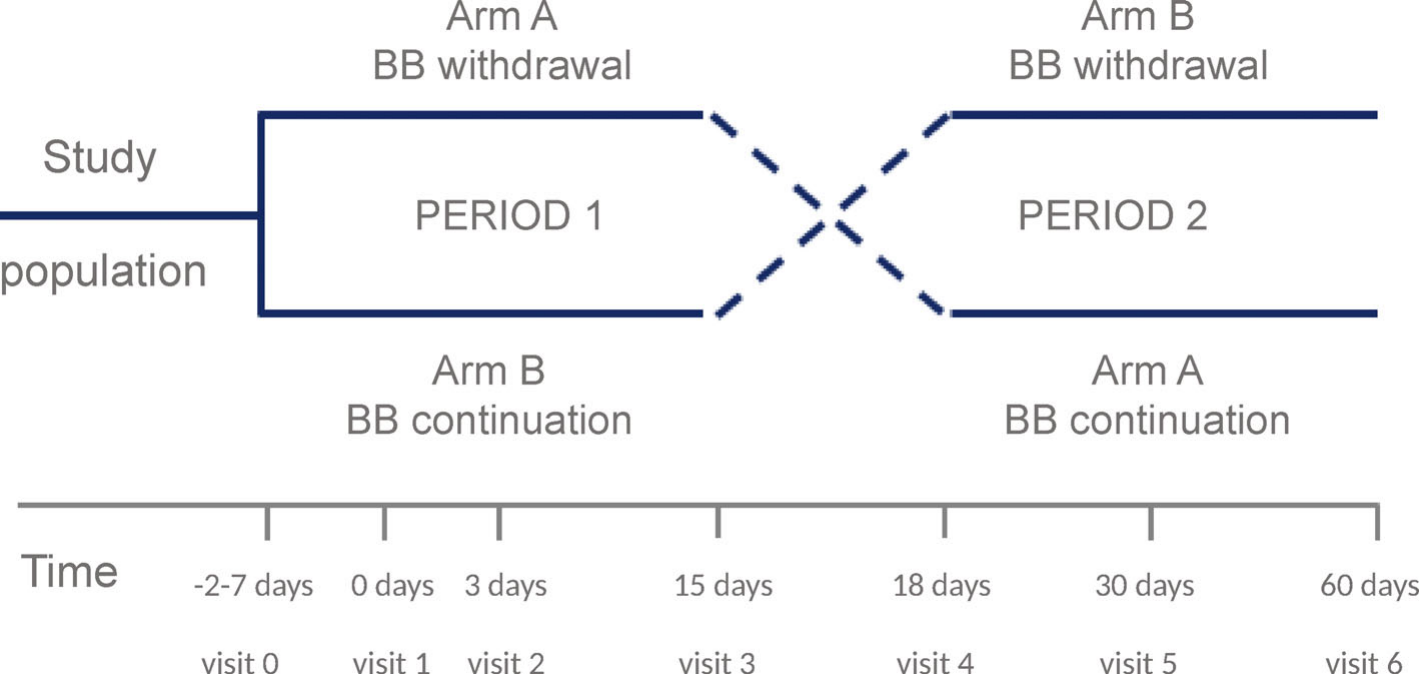
Flow chart for patient inclusion and follow up. BB, beta‐blockers

This study is designed as a multicenter, prospective, controlled, randomized, two‐arms, cross‐over, efficacy trial. The population includes patients with the diagnosis of stable HFpEF according to criteria of the European Society of Cardiology^18^ and New York Heart Association functional (NYHA) class II‐III/IV. A computer‐generated randomization sequence previously designed will be used to allocate participants (in a 1:1 ratio) to receive: (a) withdrawal of beta‐blocker followed by beta‐blocker reintroduction in two periods of 15 days; or (b) continuation of beta‐blocker followed by beta‐blocker withdrawal in two periods of 15 days. A summary of the study design is described in Figure 1. The study will be conducted in two centers in Spain. Discounting the time due to staggered entry, the total duration of a patient's follow‐up will be 6 months. All patients will provide signed informed consent before randomization. The research ethics committee approves the protocol of our center following the principles of the Declaration of Helsinki and national regulations.

### 2.2 Study population sampling

Candidate patients will be selected from the outpatient HF‐clinics of the Hospital Clínico Universitario of Valencia‐Spain and Hospital Clínico Universitario de Santiago‐Spain. Treatment will be following current guidelines^18^ and HF educational programs of each institution. Briefly, HFpEF is defined as the presence of: (a) symptoms and signs of HF with normal (≥50%) left ventricular ejection fraction (LVEF); (b) elevation of natriuretic peptides; and (c) increase in LV wall thickness and/or increased left atrial (LA) size as a sign of increased filling pressures. Inclusion and exclusion criteria are summarized in Table 1. Inclusion criteria require the presence of ChI after performing the cardiopulmonary exercise testing (CPET). The chronotropic index is equal to (heart rate at peak exercise−resting heart rate)/([220 – age] − resting heart rate).^19^ ChI is defined as a chronotropic index <0.62.

**Table 1:** Inclusion and exclusion criteria

Inclusion criteria	Exclusion criteria
Stable symptomatic heart failure (NYHA functional class ≥II) during the last month.	Inability to perform a valid baseline exercise test
Diagnosis criteria of HFpEF according to ESC guidelines: (a) symptoms and signs of HF (b) left ventricular ejection fraction >50% by Simpson method (c) NT‐proBNP >125 pg/mL in the last month (d) at least one additional criterion: 1. relevant structural heart disease (LVH and/or LAE); and/or 2. diastolic dysfunction	Significant primary pulmonary disease; including pulmonary arterial hypertension, chronic thromboembolic pulmonary disease or chronic obstructive pulmonary disease
	Patient with prior history of left ventricular ejection fraction <50%
	History of an acute coronary syndrome in the previous 12 months
Adults >18 years old	Effort angina or signs of ischemia during CPET
Previous admission for acute heart failure	Significant primary moderate to severe valvular disease
Previous treatment with beta‐blockers during the last 3‐month	Any other comorbidity with a life expectancy lower than 1 year
	Chronic treatment with digitalis or calcium channel blockers
Chronotropic incompetence assessed by CPET, defined as: [(HRmax − HRrest)]/[(220 − age) − (HRrest)] < 0.62	HR at rest >75 bpm
	Uncontrolled blood pressure, defined as systolic blood pressure > 140 mmHg and/or diastolic blood pressure > 90 mmHg.

Abbreviations: CPET, cardiopulmonary exercise testing; ESC, European Society of Cardiology; HF, heart failure; HFpEF, heart failure with preserved ejection fraction; HRmax, heart rate at maximum effort; HRrest, heart rate at rest; LAE, left atrial enlargement; LVH, left ventricular hypertrophy; NT‐proBNP, N‐terminal prohormone of brain natriuretic peptide; NYHA, New York Heart Association.

### 2.3 Intervention

#### 2.3.1 Eligibility assessment and screening visit

After reviewing the inclusion/exclusion criteria and signing the informed consent form, a comprehensive medical history, physical examination, anthropometry, and examination tests will be performed by two blinded cardiologists to patients' allocation groups. The examination tests will include an electrocardiogram (ECG), two‐dimensional transthoracic echocardiography, CPET, cognitive assessment by MMSE and MoCa, QoL assessment by MLHF, continuous ECG recording during the first 30‐day and blood samples for a panel of baseline biomarkers.

Finally, if the patients fulfill all the inclusion criteria (including ChI assessed by CPET) and any exclusion criteria (including a valid CPET without signs of ischemia), are randomized in a 1:1 ratio to one of the following interventions: (a) A‐arm: beta‐blockers withdrawal; or (b) B‐arm: beta‐blockers continuation.

#### 2.3.2 Treatment intervention and visits

Following screening (visit 0) and randomization (visit 1) visits, the procedures across treatment arms are:

*‐A‐arm*: Patients allocated to this arm are instructed to reduce by half the dose of beta‐blocker (Figure 1). The patients will be advised for potential adverse effects and instructed to contact with outpatient's clinics of HF if any adverse effect occurs. Patients will be checked in 3 days (visit 2) by a cardiologist. If clinically stable, the patients are instructed to withdraw the beta‐blocker and repeat all the procedures of the study at 15 days (visit 3). After visit 3, the patients initiate the previous half dose of beta‐blockers. Patients are visited at 18 days (visit 4). If clinically stable, the patient will increase to the previous dose of the beta‐blocker and repeat all the examination tests at 30 days (visit 5).

*‐B‐arm*: Patients allocated to this arm will continue with the same treatment and revisited 3 days after (visit 2) (Figure 1). All of the study procedures repeated at 15 days (visit 3), and, after them, the patients are instructed to reduce by the half dose of beta‐blocker. Likewise, the patients are advised for potential adverse effects and instructed to contact outpatient HF‐clinics of HF if any adverse effect occurs. Patients are revisited in 3 days (at 18 days, visit 4), and if clinically stable, the patient withdraws the beta‐blocker and repeats all the examination tests at 30 days (visit 5).

At visit 5, the responsible cardiologist will assess all the examination test and individually decide the convenience of beta‐blockers reintroduction or withdrawal in both arms. A cardiologist of the HF‐unit will clinically evaluate all patients at 60‐day after randomization (visit 6). Additional visits will be permitted according to the patient's clinical status and will be registered.

### 2.4 Study procedures

A scheme of procedures along the visits is presented in Figure 1.

#### 2.4.1 Cardiopulmonary exercise testing

Maximal functional capacity is evaluated with an incremental and symptom‐limited cardiopulmonary exercise testing (CORTEX Metamax 3B) on a bicycle ergometer, beginning with a workload of 10 W and increasing gradually in a ramp protocol at 10‐W increments every 1 minute. We define maximal functional capacity when the patient stops pedaling because of symptoms, and the respiratory exchange ratio (RER) is ≥1.05. During exercise, patients will be continuously monitored with 12‐lead electrocardiogram and blood pressure measurements every 2 minutes. Gas exchange data and cardiopulmonary variables are averaged every 10 seconds values. PeakVO2 is considered the highest value of VO2 during the last 20 seconds of exercise. The VE/VCO2 slope is determined by measuring the slope across the entire course of exercise.^20^ Each subject will undergo three tests (at baseline, 15 days, and 30 days).

#### 2.4.2 Echocardiography

Two‐dimensional Doppler echocardiogram is performed under resting conditions. Each subject will undergo three examinations (at baseline, 15 days, and 30 days). All parameters, including tissue Doppler parameters, are measured according to current guidelines of the European Society of Echocardiography.^21^

#### 2.4.3 Cognitive assessment by MMSE and MoCa

MMSE and MoCa tests will be used to assess the cognitive abilities and evaluate the impact of the intervention on cognitive function.^22^ Each subject will undergo three tests (at baseline, 15 days, and 30 days). Overall scores will be analyzed.

#### 2.4.4 Health‐related QoL

MLHF questionnaire^23^ will be used to assess the impact of the intervention on QoL. Each subject will undergo three tests (at baseline, 15 days, and 30 days).

#### 2.4.5 Continuous ECG recording

The heart rhythm and rate are continuously recorded during 30 days by remote monitoring systems integrated into clothing (Nuubo Suite License).

#### 2.4.6 Serum biomarkers

Three blood samples (at baseline, 15 days, and 30 days) are collected under standardized conditions for biomarkers' profiling. Prognostic biomarkers in HF will be analyzed,^24^ NT‐proBNP and CA125 will be measured by electrochemiluminescence immunoassay.

### 2.5 Sample size calculation

The null hypothesis of the study is that the mean peakVO2 absolute differences from baseline to 15 days after the withdrawal of beta‐blockers will be similar. The sample size determination for this study assumes two‐sided testing at the 0.05 significance alpha level. Because this is a randomized clinical trial, we assume no differences in peakVO2 at baseline among the two arms. Based on a prior study of our group in HFpEF, we assume eligible patients will have a mean (SD) peakVO2 of 10 ± 2.8 mL/kg/min.^8^ Along the same line, and based on prior studies about the deleterious effects of heart rate slowing in HFpEF patients, we speculate a blocker withdrawal will increase peak VO2 about 10%. With this data in mind, we assume a mean change of 1.2 mL/kg/min and a common SD of 2.0,^8^, ^25^, ^26^ a clinical meaningful change according a recent HFA position paper that consider significant clinical changes of peakVO2 those greater than 6% when baseline peakVO2 is lower than 14 mL/min/1.73 m^2^.^27^

Assuming an allocation ratio of 1:1, a total of 42 patients (21 patients per group) would provide 90% of power at a significance alpha level < 0.05. Assuming 20% of withdrawals or losses to follow‐up, a total of 26 patients per arm (52 patients) will be enrolled. The software used for sample size calculation was “xsampsi” from Stata 14.1.

### 2.6 Statistical plan

Continuous variables will be presented as mean ± SD or median (interquartile range—IQR) as appropriately; categorical variables as percentages. All statistical comparisons will be made under the intention‐to‐treat principle. A repeated‐measures analysis of variance (ANOVA) will be used for the comparisons of continuous outcomes among the two‐intervention groups. The interaction group*time‐points will be tested to unveil any effect of time (15 and 30 days) on the magnitude of the intervention. Only in the event of imbalance in baseline characteristics, repeated‐measures analysis of covariance (ANCOVA) will be used. A two‐sided *P*‐value of <0.05 will be considered to be statistically significant for all analyses. All analyses will be performed with Stata 14.1.

## 3 RESULTS: CURRENT STATUS

The ethics committee approved the protocol of our center, following the principles of the Declaration of Helsinki and national regulations. The protocol is registered at EudraCT (2017‐005077‐39) and http://clinicaltrials.gov (NCT03871803). Patients started enrolling in October 2018. As of January 14th, 2020, a total of 28 were enrolled. We expect to finish the inclusion at the end of July 2020.

The median (IQR) age of patients included is 74.3 (68‐77) years, 19 (67.9%) are women, and 28 (100%) were previously admitted for acute heart failure. Median (IQR) of NT‐proBNP is 922 (397‐2016) pg/mL.

## 4 DISCUSSION

### 4.1 Background and rationale

HFpEF is a complex and heterogeneous clinical syndrome characterized by exercise intolerance, markedly reduced functional capacity,^28^, ^29^ normal left ventricular ejection fraction (>50%), and evidence of diastolic dysfunction and left atrial enlargement.^18^ Despite being a contemporary challenge, the pathophysiological mechanisms of impaired exercise capacity and poor quality of life in these patients are not yet entirely clarified.^30^, ^31^ Among cardiac mechanisms, ChI has been proposed as a pathophysiologic mechanism associated with poorer exercise capacity in a subgroup of patients with HFpEF.^6^, ^7^, ^8^ Along this same line, recent evidence has shown that ChI is frequently present (ranging from 20% to 75%) in HFpEF patients.^5^, ^6^, ^7^, ^8^, ^9^, ^10^, ^11^

From epidemiological perspective, patients with HFpEF are usually older, predominantly females and with high prevalence of other cardiovascular comorbid conditions such as atrial fibrillation, hypertension, and renal dysfunction^1^, ^2^, ^3^, ^4^ which in the end contribute to reduced exercise tolerance, and may explain the high proportion (ranging from 50% to 60%) of beta‐blockers prescription in HFpEF patients.^3^, ^4^, ^14^ Nevertheless, emerging evidence suggests that pharmacological heart rate lowering is not beneficial in patients with preserved ejection fraction.^15^, ^16^, ^17^ In this regard, the proposed pathophysiological mechanism of pharmacological heart rate lowering in HFpEF patients is the prolongation of the filling of the cardiac chambers, which increases filling pressures, left ventricular diastolic wall stress and central arterial pressures.^12^

To date, there is no convincing evidence to support the beneficial effects of beta‐blockers prescription in HFpEF patients.^32^, ^33^, ^34^, ^35^, ^36^ A recent meta‐analysis suggests a clinically beneficial effect of beta‐blockers in patients with HF and left ventricular ejection fraction ≥40%; however, the evidence to those with left ventricular ejection fraction >50% is limited.^37^ Recently, a recent secondary study from TOPCAT showed that for patients with an EF of 50% or greater, beta‐blocker use was associated with an increased risk of HF hospitalizations but not CVD mortality.^15^ However, no prior randomized clinical trial has explored the effects of beta‐blocker withdrawal on functional capacity in HFpEF patients with documented ChI.

### 4.2 Biological plausibility

Currently, no study has evaluated the acute hemodynamic effects of beta‐blockers in HFpEF.^17^ However, clinical experience with these agents provides some insights. In this sense, some authors have suggested that prolonged diastolic filling related to heart rate lowering increases ventricular pressures in HFpEF patients.^17^, ^38^ Interestingly, beta‐blocker cessation would translate into a reduction in end‐diastolic pressures, as has been recently suggested with a decrease in natriuretic peptides following beta‐blockers withdrawal.^38^

Another potential beneficial effects of beta‐blockers withdrawal on HFpEF patients with ChI stand out: (a) increase in heart rate response during exercise which may be considered as a compensatory mechanism for maintaining cardiac output in patients with significant diastolic dysfunction; and (b) amelioration of delayed memory retrieval in cognitively impaired patients,^39^ and (c) attenuation arterial central pressures at rest and during exercise.^12^

### 4.3 Feasibility and future implications

HFpEF is a heterogeneous syndrome in which no pharmacological therapy has shown promising results. Identifying those HFpEF patients with ChI could help us to characterize the different phenotypes of this syndrome and optimize medical treatment. In this regard, beta‐blockers withdrawal could represent a treatment option in those patients with documented ChI. This strategy is an attempt to move forward into precision medicine in HFpEF by identifying the ChI phenotype and treating it accordingly.

## 5 CONCLUSION

To date, there is no evidence about the benefit of beta‐blockers in HFpEF patients, even less in those with documented ChI. In this randomized controlled trial, we aim to evaluate the effects of beta‐blocker withdrawal on short‐term functional capacity in stable HFpEF patients.

## FUNDING

This work was supported by grants from the Ministry of Economy and Competitiveness, Instituto Carlos III (PI17/01426) cofounded with EDRF founds and CIBER Cardiovascular (16/11/00420 and 16/11/00226).

## CONFLICT OF INTEREST

The authors declare no potential conflict of interests.

## Supporting information


**Appendix** S1. Supporting Information

**Appendix** S2. Supporting Information
