# Supplementary material for: Beta‐blockers withdrawal in patients with heart failure with preserved ejection fraction and chronotropic incompetence: Effect on functional capacity rationale and study design of a prospective, randomized, controlled trial (The Preserve‐HR trial)
Source: Clin Cardiol. 2020 Feb 19;43(5):423–9. doi: 10.1002/clc.23345 (PMC7244302; doi:10.1002/clc.23345)
Supplement: Supplementary file 1 — Appendix S1. Supporting Information [file CLC-43-423-s001.doc]

**Supplemental Appendix**

**Preserve-HR trial investigators:**

Patricia Palau MD, Julia Seller MD, Eloy Domínguez MD, Inés Gómez MD, Jose María Ramón, Clara Sastre, Rafael de la Espriella MD, Enrique Santas MD, Gema Miñana MD, Francisco J. Chorro MD, Jose Ramón González-Juanatey MD, and Julio Núñez MD.

**Study organization:**

- ***Executive or Steering comitee:*** Julio Núñez MD, Patricia Palau MD, Eloy Domínguez MD.
- ***Data Safety Monitoring comitee:*** Jose María Ramón, Clara Sastre, Gema Miñana MD, Rafael de la Espriella MD and Jose Ramón González-Juanatey MD.
- ***Clinical Endpoint comitee:*** Eloy Domínguez MD, Patricia Palau MD, Julia Seller MD and Inés Gómez MD.
- ***Core Laboratories:***
- *Echocardiography Laboratory:* Eloy Domínguez MD, Enrique Santas MD.
- *Cardiopulmonary Exercise Testing Laboratory:* Eloy Domínguez MD, Patricia Palau MD.
- Quality of life and cognitive assesment: Julia Seller MD, Patricia Palau MD.

**Ethics Committee Members**

Marina Soro MD, Maria Jesús Puchades MD.

Fundación de Investigación del Hosputal Clinico de Valencia INCLIVA

**Data Monitoring Committee Members**

Ana Portolés, Marta Peiró.

Fundación de Investigación del Hospital Clínico de Valencia INCLIVA

**Clinical Events Committee Members**

Antoni Bayés-Genís MD and Lorenzo Fácila MD
