# Supplementary material for: Beta‐blockers withdrawal in patients with heart failure with preserved ejection fraction and chronotropic incompetence: Effect on functional capacity rationale and study design of a prospective, randomized, controlled trial (The Preserve‐HR trial)
Source: Clin Cardiol. 2020 Feb 19;43(5):423–9. doi: 10.1002/clc.23345 (PMC7244302; doi:10.1002/clc.23345)
Supplement: Supplementary file 2 — Appendix S2. Supporting Information [file CLC-43-423-s002.doc]

**SUPPLEMENTAL MATERIAL**

**SUPPLEMENTAL METHODS**

**Procedures**

*Cardiopulmonary exercise testing*

Maximal functional capacity will be evaluated with an incremental and symptom-limited cardiopulmonary exercise testing (CORTEX Metamax 3B) on a bicycle ergometer, beginning with a workload of 10 W and increasing ramp at 10-W increments every 1 min. During exercise, patients will be continuously monitored with twelve-lead electrocardiogram and blood pressure measurements every 2 min. Gas exchange data and cardiopulmonary variables will be averaged every 10 seconds. Peak oxygen uptake (peakVO2) will be considered the highest value of VO2 during the last 20 s of exercise. The percent of predicted peakVO2 (pp-peakVO2) will be calculated using the Wasserman equation [1]. The ventilatory efficiency (VE/VCO2 slope) will be determined by measuring the slope across the entire course of exercise [2]. Physical effort will be assessed by respiratory exchange ratio (RER). Oxygen pulse will be also evaluated.

Heart rate response during effort will be evaluated according to chronotropic index formula. The chronotropic index is equal to: (heart rate at peak exercise−resting heart rate)/[(220−age)−resting heart rate] [3]. Chronotropic incompetence is defined as a chronotropic index < 0.62.

Each patient will undergo 3 cardiopulmonary exercise testing: a) the first one at baseline visit (visit 1); b) second one at visit 3; and c) third one at visit 5. The impact of the intervention on functional capacity will be evaluated by absolute and relative changes in peakVO2 and pp-peakVO2.

*Echocardiography*

Doppler echocardiogram examinations will be performed under resting conditions using 2D echocardiography (iE33, Philips). All parameters, including tissue Doppler parameters will be measured according to current guidelines of the European Society of Echocardiography [4].

Each patient will undergo 3 echocardiographs: a) the first one at baseline visit (visit 1); b) second one at visit 3; and c) third one at visit 5. The impact of the intervention on diastolic function and left atrial volumes will be evaluated by changes in echocardiography parameters of diastolic function and left atrial volumes.

*Continuous ECG recording*

The heart rhythm and rate are continuously recorded during intervention (30 days) by remote monitoring systems integrated into clothing (Nuubo Suite Licence). Maximum, median and minimum heart rate during remote monitoring will be analyzed for security reasons.

*Health related quality of life*

Health related quality of life will be evaluated with the by the Minnesota Living With Heart Failure Questionnaire (MLHF) [5]. Each subject will undergo 3 tests (at baseline, 15-day, and 30-day) Overall scores will be analyzed. The impact of the intervention on quality of life will be evaluated by changes in the score.

*Cognitive assessment by MMSE and MoCa*

Cognitive function will be assessed by the Mini-Mental State Examination (MMSE) and Montreal Cognitive Assessment (MoCa) tests [6]. Each subject will undergo 3 tests (at baseline, 15-day, and 30-day) Overall scores will be analyzed. The impact of the intervention on cognitive function will be evaluated by changes in the scores.

**REFERENCES**

[1] Wasserman K. Principles of exercise testing and interpretation: including pathophysiology and clinical applications. Philadelphia: Lippincott Williams & Wilkins. 2005.

[2] Arena R, Myers J, Aslam SS, Varughese EB, Peberdy MA. Technical considerations related to the minute ventilation/carbon dioxide output slope in patients with heart failure. Chest. 2003; 124: 720–727.

[3] Brubaker PH, Kitzman DW. Chronotropic incompetence: causes, consequences and management. Circulation 2011; 123: 1010– 1020.

[4] Paulus WJ, Tschöpe C, Sanderson JE, et al. How to diagnose diastolic heart failure: a consensus statement on the diagnosis of heart failure with normal left ventricular ejection fraction by the Heart Failure and Echocardiography Associations of the European Society of Cardiology. Eur Heart J. 2007; 28: 2539-50.

[5] Rector TS, Cohn JN. Assessment of patient outcome with the Minnesota Living with Heart Failure questionnaire: reliability and validity during a randomized, double-blind, placebo-controlled trial of pimobendan. Pimobendan Multicenter Research Group. Am Heart J. 1992;124 : 1017-25.

[6] Hawkins MA, Gathright EC, Gunstad J, et al. The MoCA and MMSE as screeners for cognitive impairment in a heart failure population: a study with comprehensive neuropsychological testing. Heart Lung. 2014;43(5):462-8.
